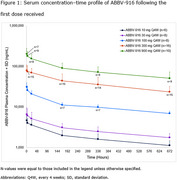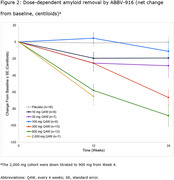# A randomized, double‐blind, placebo‐controlled, multiple ascending dose study to evaluate the efficacy, pharmacokinetics, and safety of ABBV‐916 in adults with early Alzheimer’s disease

**DOI:** 10.1002/alz70859_102775

**Published:** 2025-12-25

**Authors:** Shau Yu Lynch, Yamin Wang, Deli Wang, Sagar Bachhav, Hao Xiong, Joey Boiser, Edwin Stage, Anthony W. Bannon, Ole Graff, Hana Florian

**Affiliations:** ^1^ AbbVie Inc., North Chicago, IL USA

## Abstract

**Background:**

Alzheimer’s disease (AD) is a progressive, irreversible, fatal neurodegenerative disorder, and is the leading cause of dementia among the elderly population globally. ABBV‐916 is a potential disease‐modifying treatment for AD that targets Aβ peptides bearing a pyroglutamate residue at amino acid position 3, an Aβ species predominantly found in parenchymal amyloid plaques. This study aims to evaluate the efficacy, pharmacokinetics (PK) and safety of ABBV‐916 in participants with early AD.

**Method:**

This Phase 1b/2, multicenter, randomized, placebo‐controlled, multiple ascending dose study included adults aged 50–90 years, with Stage 3 or 4 AD, a Mini‐Mental State Examination score of 20–28, and a positive amyloid positron emission tomography (PET) scan ≥37 centiloids. Six cohorts with doses ranging from 10–3,000 mg were planned. Participants were randomized 3:1 to receive either ABBV‐916 or placebo every 4 weeks (Q4W) intravenously over a 24‐week double‐blind period. Safety monitoring included adverse events (AE), clinical laboratory assessments, and magnetic resonance imaging scans. Dose escalation occurred only after the review of safety, PK and PET assessments (where available) by the safety committee.

**Result:**

Preliminary results included 106 patients receiving doses from 10–2,000 mg, of which 74 had ≥1 post‐baseline PET scan. After 24 weeks, PK exposures (maximum observed serum concentration [C_max_] and area under the curve [AUC_0‐D29_]) were dose proportional across all doses, with low inter‐subject variability (Figure 1). Treatment with ABBV‐916 300 mg and 900 mg resulted in a dose‐dependent brain amyloid reduction from baseline (Figure 2), with 60.0% and 85.7% of participants, respectively, achieving amyloid negativity at Week 24.

Across all cohorts, AEs and serious AEs were reported by 61.3% and 6.6% of participants, respectively, and 12.3% of participants discontinued treatment due to AEs. No deaths occurred during the double‐blind period. The incidences of amyloid‐related imaging abnormalities‐edema (ARIA‐E) and hemosiderin deposition (ARIA‐H) were 19.8% and 17.0%, respectively, and infusion‐related reactions occurred in 6.6% of participants.

**Conclusion:**

Preliminary data suggest that the safety and PK profile of ABBV‐916 is comparable to currently approved anti‐amyloid immunotherapies for the treatment of AD. Brain amyloid reduction was observed at doses of 300 mg or higher.